# Childhood TB Surveillance: Bridging the Knowledge Gap to Inform Policy

**DOI:** 10.1155/2012/865436

**Published:** 2012-02-14

**Authors:** Andrew J. Brent

**Affiliations:** ^1^KEMRI-Wellcome Trust Research Programme, P.O. Box 230, Kilifi 80108, Kenya; ^2^Wellcome Trust Centre for Clinical Tropical Medicine, Imperial College London, London W2 1PG, UK; ^3^Department of Pediatrics, Imperial College London, London W2 1PG, UK

## Abstract

Tuberculosis (TB) is a leading cause of death globally. Natural history studies show that young children are at particularly high risk of progression to active TB and severe, disseminated disease following infection. Despite this, high-quality regional and global surveillance data on the burden of childhood TB are lacking. We discuss the unique aspects of TB in children that make diagnosis and therefore surveillance challenging; the limitations of available surveillance data; other data which provide insights into the true burden of childhood TB. Improved surveillance is among the key research priorities identified for childhood TB, but progress to date has been slow. Recent advances in TB diagnostics, and standardized clinical diagnostic guidelines and case definitions, all provide opportunities for new strategies to improve surveillance. Better-quality data on the burden and trends of childhood TB will inform and improve both public health policy and clinical practice.

## 1. Introduction

Three main challenges currently hamper efforts to control tuberculosis (TB) globally. Firstly, the ability to identify and diagnose cases remains suboptimal and is particularly poor among children and human-immunodeficiency-virus- (HIV-) infected individuals, with direct implications for the availability and quality of surveillance data in these groups. Secondly, there is a need for better (particularly shorter) treatment regimens to improve individual case management and to prevent and control the emergence and spread of drug resistant strains. Thirdly, the search is still on for better tuberculosis vaccines to provide more complete and longer-lasting protection against active TB. Success in all three areas is likely to be required to achieve the Stop TB goal to eliminate TB as a public health problem by 2050 [1, 2]. This paper addresses the first of these challenges and focuses on the need and potential strategies to improve diagnosis and thereby surveillance of childhood TB in low-resource, high-burden settings.

**Figure 1:**
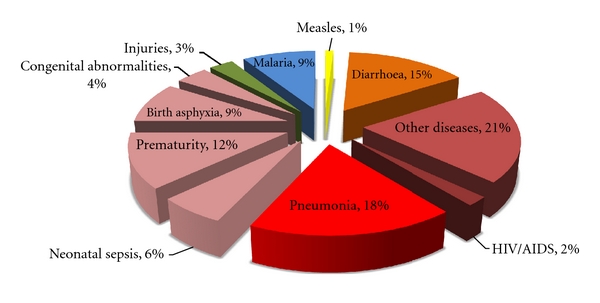
Causes of death among children under 5 years of age (data from [6]).

In 2009 an estimated 9.4 million cases of TB occurred worldwide, equivalent to an annual global incidence of 137 per 100,000 population [3]. In the same year, 1.7 million deaths were attributed to tuberculosis [3]. TB remains second only to HIV as a leading infectious cause of death globally [4]. Global estimates of the causes of death among children have also been derived [5, 6], and the latest estimates based on data from 2008 are illustrated in Figure 1 [4]. Given the overwhelming burden of tuberculosis among the population as a whole and the well-documented vulnerability of young children to active TB including the severe and fatal forms of the disease [7], it is perhaps surprising that TB does not feature among the leading causes of death in children. Protection afforded by Bacille Calmette Guérin (BCG) vaccination among infants and young children, particularly against disseminated TB and TB meningitis, provides one important explanation for this apparent discrepancy [8]. However, even in this group the protective efficacy of BCG is suboptimal, and the best available estimates suggest a high global burden of childhood TB despite wide-scale neonatal BCG vaccination within the Expanded Programme of Immunization [9]. To reconcile these data requires a clear understanding of their limitations and the underlying challenges of TB diagnosis in children.

## 2. Diagnosis of Childhood TB and Implications for Surveillance

The pathophysiology, clinical presentation, and investigation of TB in children differs from that in adults due to a combination of immunological, anatomical, and epidemiological factors, all of which make diagnosis considerably more challenging [10]. Young children are at much higher risk of progression to active TB following infection [7]. Larger inocula due to prolonged close contact with adults in the household from whom they frequently acquire their infection might contribute to this higher risk [11, 12], but age-related differences in both innate and adaptive immune responses to TB are thought to be the most important determinants of their increased vulnerability [13]. Although poorly understood, a less robust immune response is also likely to underlie the paucity of lung cavitations in young children with TB, in stark contrast with the classic picture of adult pulmonary TB in which large numbers of bacilli may “spill” from the cavities into the airways. Whatever the exact mechanisms, important consequences of these differences in host immunity include relatively fewer bacilli, particularly in clinical specimens (“paucibacillary” disease), and an increased propensity for disseminated, extra-pulmonary disease in young children [10].

This in turn has implications for diagnosis since specimen collection is often more difficult from extra-pulmonary sites, especially in low-resource settings, and low numbers of bacilli greatly reduce the sensitivity of both smear microscopy and mycobacterial culture. Anatomical differences limit access to appropriate respiratory specimens in childhood pulmonary TB, too, since smaller airways and less tussive force limit a child's ability to expectorate sputum, necessitating more invasive and labour intensive methods of specimen collection such as sputum induction or gastric aspiration [14]. Immune-based diagnosis using tuberculin skin testing (TST) or newer interferon gamma release assays (IGRAs) is limited by poor sensitivity and specificity [15]. Furthermore, although they are recommended components of the TB diagnostic workup for children where available, in practice TST, gastric lavage and sputum induction are often not available in high-burden settings due to limited training, a lack of the basic equipment and consumables required [16, 17], very low yield in the absence of mycobacterial culture facilities [14], and cost [18, 19]. IGRAs remain far too expensive and complex for use at the point of care in low-resource settings.

In the absence of a reliable gold standard and lacking resources for further investigations in most settings, diagnosis therefore usually has to rely on clinical case definitions. To compound the problem, clinical diagnosis is also more challenging in children, since the clinical and radiological features of childhood TB overlap those of other common childhood diseases, particularly where HIV and malnutrition—two of the most important risk factors—are endemic [20]. Several clinical algorithms have been proposed, but although they continue to be widely promoted, most have not been validated in the settings in which they are used [21]. The net result is frequent disease misclassification that severely limits the quality of both clinical care and surveillance data.

## 3. Available Surveillance Data and Burden of Disease Estimates

The main source of global surveillance data for childhood TB comes from notification data collected by National Tuberculosis Programmes (NTPs) under the WHO DOTS strategy [3]. However, both the quantity and the quality of DOTS notification data relating to childhood TB are limited. Until recently data from children were aggregated in a single category (<15 years) [22], ignoring important age-related differences in disease incidence between preschool, school age, and adolescent children. This reflected the lower priority traditionally afforded to children by the DOTS strategy, which understandably prioritizes detection and treatment of smear positive pulmonary cases that contribute most to propagation of the epidemic but occur predominantly in adults. One consequence is a lack of data on age-related trends among children that might help refine burden of disease estimates or afford additional insights into the effects of the HIV epidemic or other epidemiological risk factors on disease presentation or transmission patterns. The WHO recommendation in 2006 for NTPs to report aggregated data for children in at least two categories (0–4 years and 5–14 years) was a welcome step in the right direction [19]. Nevertheless, severe limitations still exist in the quality of available TB notification data for children due to frequent misclassification arising from the very real diagnostic challenges highlighted above, a lack of resources for active case finding through contact tracing in most settings, as well as limitations in the notification process itself. A recent study from South Africa demonstrated significant undernotification of TB cases [23], and even in Europe 1 in 5 children with TB are not notified [24], so the situation is likely to be considerably worse in less well-resourced settings. Perhaps reflecting all these problems with the available data, WHO estimates of the global TB burden do not currently include a breakdown of the disease burden in children [3].

To date the best available published estimates of the global burden of childhood TB have been derived by combining DOTS notification data for smear positive TB with age-specific estimates of the proportion of the total number of cases that are smear positive [9, 25]. These estimates suggest there were approximately 900,000 new cases of TB in children in 2000, which represented about 11% of the total global burden [9]. These are crude estimates, however, due to the inherent limitations of the data on which they are based. Even the proportion of childhood TB cases which one might expect to be smear positive (on which these estimates rely) is not straightforward to ascertain in the absence of a reliable gold standard for diagnosis since it requires knowledge of the total number of childhood cases in a particular setting; furthermore, this proportion may vary geographically or temporally with the prevalence of HIV infection, malnutrition, or other risk factors.

Better-quality surveillance data are available from many industrialized, low-burden settings [26, 27], where they are underpinned by stronger health systems with better diagnostic facilities and active case finding through routine contact tracing. Burden of disease estimates from some high-burden communities are also available and suggest that children may account for approximately 20% of the total caseload in such settings [28]. In one frequently cited study based on official notification data from two urban settings in Cape Town, South Africa, children accounted for almost 40% of the total caseload over a 10-year period [29]. In the absence of a standardized case definition in this study, it is unclear whether this reflects the true potential burden of childhood TB in settings where deteriorating socioeconomic conditions drive high TB transmission rates, or a tendency towards overdiagnosis by clinicians aware of the high TB incidence combined with the poor performance of available diagnostic tools and the potentially devastating consequences of not treating a child with TB. A prospective observational study of childhood TB in a very similar urban setting in Cape Town suggested children accounted for about 15% of all TB cases [30]. However, the number of published studies is small, and they derive from a minority of countries that may not be representative of the overall picture.

The need for better-quality surveillance data from different communities and underpinned by improved case definitions was therefore recognized as among the leading research priorities for childhood TB in the WHO's *Research Agenda for Childhood Tuberculosis* [28]. There has been very limited progress in this area, however. A PubMed literature search performed on 14 October 2011 using the MeSH and text search terms “tuberculosis” or “TB,” plus “child” or “children” and “incidence” or “prevalence” identified only 15 studies presenting population-based data on the disease burden of childhood TB since the report's publication 5 years befor: 5, 8, and 2 from high- [26, 27, 31–33], middle- [34–41] and low- [42, 43] income countries, respectively. There were no studies from any low- /middle-income or high-TB-burden countries [3] that met the basic criteria for prospective community incidence studies set out in the WHO report [28].

In the absence of good-quality surveillance data, other data sources may also be used to inform burden of disease estimates. Historical data from Europe correlating the incidence of childhood TB disease with the annual rate of tuberculosis infection (ARTI, derived from tuberculin surveys) has been extrapolated to estimate childhood TB incidence in modern communities in whom the population structure and ARTI is known [44]. Interesting data also come from pneumonia aetiology studies among children, some of which have found active TB in 8–15% of children with acute pneumonia [45, 46]. This may be particularly pertinent to the question of hidden childhood TB mortality, since pneumonia is responsible for the largest proportion of deaths in children under 5 years (Figure 1). Autopsy data are scarce, but a study in Zambia prior to the availability of antiretroviral therapy or widespread use of cotrimoxazole prophylaxis found active TB in 26% HIV-negative and 18% HIV-positive children who died from pneumonia [47]. These findings call for similar studies in other settings to investigate their generalizability.

## 4. Opportunities and Strategies for the Future

More robust estimates of childhood TB incidence and its contribution to childhood mortality will depend critically on improved surveillance in a wide range of settings. Rigorous implementation of existing guidelines for contact investigation among children [19] would improve active case detection as well as providing real clinical benefit through identification or earlier diagnosis and treatment of many cases. Diagnosis remains the fundamental challenge, but recent progress in this area provides opportunities to improve case ascertainment and reduce disease misclassification. Standardized case definitions are key, so the publication by the WHO of guidance on the investigation and diagnosis of childhood TB including case definitions for smear positive and negative pulmonary disease is an important step [19]. Further research to build on these case definitions and to incorporate them into validated diagnostic algorithm(s) based on high-quality evidence from a range of settings—as has been done with some success for TB in HIV-positive adults [48]—would offer benefits for both surveillance and clinical care. Consensus definitions of childhood intrathoracic tuberculosis drafted by an international panel of experts for research purposes have recently been submitted for publication (Stephen Graham, personal communication).

Ideally diagnosis should be supported by the best available methods for specimen collection and microbiological confirmation of *M. tuberculosis*. While this is unlikely to be feasible in most low-resource, rural settings, research gains slowly being translated into clinical practice do provide an opportunity to develop heightened surveillance in some centres—ideally linked to existing TB prevalence surveys or studies of TB incidence among adults, in order to place in context and maximize the scientific and public health benefits of the epidemiological data they generate.

The same sites could be used to assess and monitor the local performance of more basic clinical case definitions and diagnostic algorithms. Sputum induction has been shown to be well tolerated, safe, and more sensitive than gastric aspiration for diagnosis of pulmonary TB in children and has the added advantage that it can be performed at a single outpatient visit [14]. Although uptake of the method has been slow in high-burden settings, it is slowly being adopted in some urban centres and might be combined with gastric aspiration at the same visit to further increase TB culture yield [49]. Mycobacterial culture facilities are also required if the full benefit of improved specimen collection is to be realized, but progress is also being made on this front by the Stop TB Partnership Global Laboratory Initiative (http://www.stoptb.org/wg/gli/) [50, 51]. Although these are likely in the medium term to remain restricted to a few urban referral centres in many developing countries, other developments in laboratory diagnosis might yet offer the possibility of expanding access to high-quality microbiological diagnosis in low-resource, high-TB-burden settings.

The Microscopic Observation Drug Susceptibility (MODS) and Thin Layer Agar (TLA) microcolony culture techniques provide culture and drug susceptibility results at a fraction of the cost of commercial liquid culture systems and do not require expensive biosafety level 3 laboratory facilities, but are relatively labour intensive and require significant training [52]. More recently the Xpert MTB/RIF automated real-time PCR assay, which requires minimal training and infrastructure but is more expensive, has demonstrated modest sensitivity for diagnosis of culture positive TB in children [53]. All require further research to assess their performance and cost effectiveness in a range of settings.

The benefits of improved childhood TB surveillance are many. At a public health level, good-quality regional and global data are critical to understand the causes of childhood illness and death, thereby informing policy decisions and priorities. Disease visibility is key to ensure appropriate investment in prevention and control strategies and will also be important to monitor the impact of new TB vaccines in the future. In addition, since TB in a child must by definition reflect recent infection (within the lifetime of the child) and in most cases occurs within a year following infection [7], the incidence of TB in children and their *M. tuberculosis* isolates provide a useful window on current transmission dynamics within a community, including transmission of drug resistance strains. Benefits to clinical care might be expected due to improved case finding, standardization of case definitions, local validation, and/or adaptation of diagnostic algorithms utilizing data from sentinel sites with access to more advance diagnostic facilities, and a greater awareness of the burden and diagnostic algorithms among clinicians. Children remain among the most vulnerable to this common and all too often devastating disease. A better understanding of the size of the problem will be essential to any public health strategy to control childhood TB.
